# Fibroblasts in rheumatoid arthritis: novel roles in joint inflammation and beyond

**DOI:** 10.3389/fmed.2024.1376925

**Published:** 2025-01-21

**Authors:** Elpida Neofotistou-Themeli, Panagiota Goutakoli, Theodoros Chanis, Maria Semitekolou, Eirini Sevdali, Prodromos Sidiropoulos

**Affiliations:** ^1^Laboratory of Rheumatology, Autoimmunity and Inflammation, University of Crete, Medical School, Heraklion, Greece; ^2^Institute of Molecular Biology and Biotechnology, Foundation for Research and Technology - Hellas (FORTH), Heraklion, Greece; ^3^Division of Immunology and Allergy, Department of Medicine, Karolinska Institute, Solna, Sweden; ^4^Center for Molecular Medicine, Karolinska Institute, Stockholm, Sweden; ^5^Dendritic Cells and Adaptive Immunity Unit, Immunology Department, Pasteur Institute, Paris, France; ^6^Developmental Biology and Stem Cells, UMR3738 – National Center for Scientific Research (CNRS), Pasteur Institute, Paris, France

**Keywords:** rheumatoid arthritis, fibroblast, interstitial lung disease (ILD), synovium, lung, ACPA

## Abstract

High-throughput technologies in human and animal studies have revealed novel molecular and cellular pathways involved in tissue inflammation of rheumatoid arthritis (RA). Fibroblasts have been in the forefront of research for several decades. Subpopulations with specific phenotypic and functional properties have been characterized both in mouse models and human disease. Data supporting the active involvement of fibroblasts in immune responses and tissue remodeling processes, as well as their central role in promoting clinical relapses and contributing to treatment resistance, have clearly reshaped their role in disease evolution. The lung is an important non-synovial component of RA both from a clinical and an immunopathogenic aspect. Interstitial lung disease (ILD) is a significant contributor to disease burden affecting morbidity and mortality. Although our knowledge of ILD has progressed, significant gaps in both basic and clinical science remain, posing hurdles to efficient diagnosis, prediction of disease course and its effective treatment. The specific role and contribution of fibroblasts to this process has not been clearly defined. The focus of this review is on fibroblasts and their contribution to RA and RA-ILD, presenting data on genetics and immune responses associated with RA-ILD in humans and animal models.

## Introduction

Systemic Autoimmune Rheumatic Diseases (SARDs) are a group of immune-mediated disorders characterized by diverse clinical manifestations. SARDs exhibit continuously rising incidence in Western countries and a lifetime prevalence of over 10% in the European Union (1). They contribute substantially to morbidity, mortality and annual health care costs. Common denominators in SARD development include environmental, genetic and immune factors. They orchestrate the initiation, progression and amplification of inflammatory networks and dictate responses to therapy. Although several achievements have been accomplished during recent decades for the diagnosis and treatment of SARDs, there are still significant unmet needs which impede improvements in clinical outcomes, quality of life, and in lowering the cost of treatment.

Rheumatoid arthritis (RA) is a SARD which has a significant societal and financial burden in Europe (2). It is widely recognized that by the time of initial diagnosis of RA, as is the case for many other SARDs, the autoimmune response has already been established and chronic inflammation is mostly irreversible. RA is the most prevalent SARD that primarily affects the joints. In Western Europe, its prevalence is estimated to be 0.63% in females and 0.24% in males, while it increases significantly to up to 2% among individuals over age 60 (3). The treatment goal for RA is remission of the inflammatory process. The management of RA in clinical practice has benefited from recent advances such as early diagnosis and treatment, adoption of the “treat to target” strategy and the introduction of targeted biologic therapies. Nevertheless, a lot of improvement is still needed in disease related outcomes (functional limitation, significant morbidity and increased mortality) (4). Although recent data show that ‘hard’ outcomes like mortality have improved in some cohorts (5, 6), most epidemiological data still indicate a gap compared to the non-RA control population (7, 8). Leading causes of mortality are cardiovascular diseases and lung involvement, primarily interstitial lung disease (ILD) and infections. Clinically significant ILD occurs in approximately 10% of RA patients, while the prevalence of subclinical abnormalities consistent with ILD has been documented as up to 60% (9, 10). Inflammation and fibrosis within the lung parenchyma is the pathophysiological basis of RA-ILD, as it is in many other organs affected in various SARDs. Although clinical, epidemiological and imaging characteristics have been associated with the risk of ILD development and its outcome, they do not represent clinically reliable tools to be used for individual monitoring and clinical decision making.

Fibroblasts are cells of mesenchymal origin present in all tissues. They adopt different phenotypes and functions according to intrinsic characteristics or upon responding to different acute and chronic stimuli (11). High-throughput single-cell RNA sequencing studies in inflammatory arthritis mouse models and in the human RA synovium have revealed several fibroblastic subpopulations with specific phenotypic and functional properties, while spatial diversity has also been identified within the synovium (12–14). The active contribution of fibroblasts to immune and tissue remodeling processes, clinical relapses and treatment resistance has reshaped how we perceive the role of fibroblasts in RA evolution and outcome (15, 16). Functionally distinct subpopulations contribute to lung development, response to injury and repair (17). However, studies focusing on the pathogenesis of RA associated ILD are limited, and no clear pathophysiology concept is accepted.

This review focuses on fibroblasts, presenting recent developments pertinent to their contribution to RA and RA-ILD pathogenesis – from genetic data associated with RA-related lung disease to major discoveries in fibroblast subpopulations – and discussing their contribution to disease evolution, flares and treatment responses in human RA and in experimental models of arthritis.

## Overview of RA pathogenesis

**FIGURE 1:**
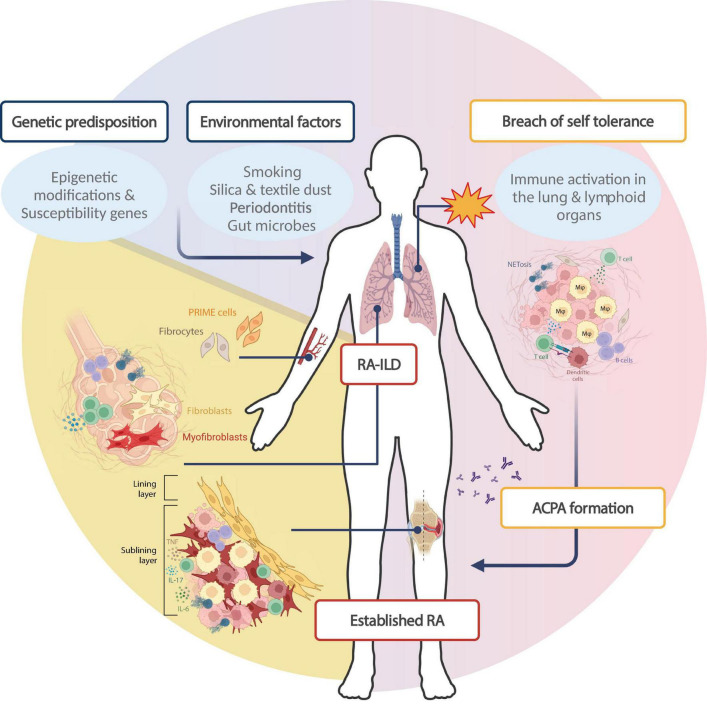
Rheumatoid arthritis pathogenesis: specific contribution of fibroblasts in RA phenotype. The paradigm for RA development stands that *environmental parameters* trigger primary autoimmune events in *genetically predisposed* individuals. This interaction leads to *loss of self-tolerance* in extra-synovial tissues like the lung and lymphoid tissues, through the activation of immune cells of innate and adaptive immunity. Protein citrullination in mucosal areas like the lung combined with RA-associated HLA-DR alleles leads to the production of *anti-citrullinated protein antibodies (ACPAs)* in 50–60% of patients. Thereafter, autoreactive immune cell homing to the synovial compartment triggers synovitis (early arthritis), characterized by local inflammation and a dramatic expansion of the synovial membrane, characterized by extensive proliferation of fibroblasts and further immune cell recruitment. *Established RA* is a systemic disease with extra-articular manifestations; approximately 10% of RA patients develop interstitial lung disease (RA-ILD), which has a complex pathogenesis implicating both immune and stromal cells. Within the lung, collagen deposition and subsequent fibrosis is driven by resident macrophages and dendritic cells which favor fibroblast and myofibroblast activation, though the fibroblast subsets involved have not been extensively studied. Of note, circulating pre-inflammatory mesenchymal (PRIME) cells have been proposed to drive disease flares, while circulating fibrocytes could constitute a pathogenic link between joint and lung pathology in RA. Created in BioRender. Neofotistou, E. (2025). https://BioRender.com/l38d140.

The identification of anti-citrullinated protein antibodies (ACPAs) set the stage to advance our understanding of RA pathogenesis. 50–60% of RA patients have ACPA antibodies, which are highly specific for RA, together with rheumatoid factors (RF), which have a lower specificity for RA (18). Most of the knowledge for disease pathogenesis refers to seropositive disease, while data for seronegative disease is much more limited. The current paradigm for RA pathogenesis is that autoimmunity develops over several years before the disease is clinically apparent (Figure 1). In genetically susceptible individuals, early self-reactivity against post-translationally modified proteins develops mostly in mucosal areas, with subsequent targeting of synovial tissue by effector T cells. Amplification of inflammatory responses along with the transition of resident stromal cells into autoaggressive effector cells converts synovitis from acute to chronic destructive joint disease (19).

The identification of ACPAs has put together the concept that genetic and environmental factors lead to breaching of tolerance to post-translationally modified proteins and to subsequent autoimmunity development. It is known that genetic polymorphisms contribute 30–60% of the overall RA risk (20). Genome-wide association studies (GWAS) data confirmed earlier genetic association studies suggesting that HLA class II alleles, specifically HLA-DRβ1 alleles containing a particular sequence in amino acid residues 71–74 of the β-chain (“shared epitope”, SE), confer the strongest risk (21, 22). Interestingly and further supporting the pathogenetic contribution of HLA-DR genes, it has been shown that proteins encoded by these alleles can bind citrullinated peptides with higher avidity than native peptides, thereby inducing T-cell activation (23, 24). GWAS analysis has revealed more than 100 additional alleles which contribute to the risk of disease development, most of them relevant to innate and adaptive immune response pathways.

Amongst the diverse environmental factors associated with RA development, smoking shows the strongest correlation (25). Smoking is considered to act as an epigenetic modifier and, combined with HLA-DR4 alleles, contributes to ACPA formation. Several human studies in individuals at risk for RA development and early RA patients support that autoimmunity to post-translationally modified proteins develops in the lung parenchyma, resulting in effector CD4 cells and autoreactive B cells (26–28). Data elucidating the mechanisms driving the homing of autoreactive CD4 cells into the synovium, in the absence of any synovium- or joint-specificity of these cells, are limited. Elegant *in vitro* and *in vivo* studies have linked the breach in T cell tolerance to cell-endogenous abnormalities present in naive T cells, which drive the differentiation program to favor the generation of effector instead of long-lived memory T cells (19). Impaired DNA repair mechanisms, which compromise telomeric function and mitochondrial fitness, are a major driver of metabolic alterations characterized by decreased glycolysis and favoring catabolic pathways, therefore transforming T cells to tissue-invasive proinflammatory effector cells (29, 30). Among other molecular mechanisms, deficient activity of the nuclease MRE11A in T cells of RA patients has been linked to premature T cell aging, tissue invasiveness and proinflammatory T cell functions (30, 31). Upon synovial localization of inflammatory responses, acute synovitis is considered to transform into a chronic process by maladaptive tissue remodeling mechanisms driven by both immune cells and synovium stromal cells, mostly fibroblasts. Several recent studies based on single-cell RNA sequencing analysis revealed functionally distinct subsets of highly activated synovial fibroblasts, which adopt proinflammatory and tissue-invasive function, and together with tissue-infiltrating myelocytes and lymphocytes contribute to tissue damage. The application of high throughput technologies has provided unprecedented information on the cellular composition of the synovial lesion (12, 14, 32). Thus, several fibroblast, macrophage, T and B cell subpopulations have been identified. Although interesting data concerning the importance of these molecular endotypes for predicting the course of early disease and response to treatment have been shown, mechanistic studies are needed to elucidate their contribution to disease pathogenesis (12, 14, 15, 32).

## Synovial fibroblasts in RA pathogenesis

The involvement of fibroblasts in RA development and progression has been extensively studied within the synovium. In the healthy joint, fibroblast-like synoviocytes (FLS) contribute to the formation of the thin synovial membrane enclosing the synovial cavity and are crucial in maintaining cartilage integrity and function by producing extracellular matrix and synovial fluid components (33–35). In the context of RA, these cells become hyperactivated and hyperproliferative, epigenetically imprinted with an aggressive phenotype that alters the joint microenvironment by producing metalloproteases that degrade the extracellular space, invading the synovial cavity and causing cartilage damage and bone erosion directly or via RANKL-mediated osteoclast activation. Moreover, FLS act as a rheostat for immune circuits (36), exacerbating inflammation by facilitating the infiltration of immune cell populations via angiogenesis and redistribution of soluble factors, expression of cell adhesion molecules and secretion of proinflammatory stimuli such as IL-6 and IL-8 (35, 37). Of note, metabolic parameters have been found to contribute to FLS activation, proliferation and aggression in arthritis (38, 39).

Based on research applying high-throughput technologies, knowledge for the phenotypically and functionally distinct subpopulations of fibroblasts in different diseases has been dramatically expanded (Tables 1, 2). Interestingly and besides the pathophysiological correlations, several clinical implications of synovial biology for RA patients are available, concerning disease prognosis, flares and response to treatments. More specifically, early RA patients with a fibroid synovial pathotype are characterized by poor responses to treatment and worse prognosis regardless of the treatment scheme used (32). Analysis of matched peripheral blood and synovial biopsy samples highlighted the importance of synovial tissue evaluation in predicting disease outcome. Indeed, synovial histology correlates with clinical parameters and may aid in early classification of patients (40). In a cohort of established RA patients receiving different biologic agents, synovial composition was able to predict treatment responses (15). More specifically, patients with abundant myeloid cell infiltration responded better to anti-IL6R treatment with tocilizumab than to anti-CD20 treatment with rituximab, while patients with fibroblast-rich synovia displayed multidrug resistance. Elegant studies support the migratory capacity of fibroblasts in humans and mouse models of RA, attributing a role in disease states and its “spread” between different joints. A recent human study identified a B cell-induced circulating population of CD45^–^CD31^–^Podoplanin^+^ pre-inflammatory mesenchymal (PRIME) cells that could predict disease flares (16), while studies in the severe combined immunodeficient (SCID) mice receiving activated human RA synovial fibroblast implants confirmed that FLS can transmigrate, spreading arthritis to unaffected joints (41).

**TABLE 1:** Murine synovial fibroblast subsets in experimental arthritis.

Subset	Defining characteristics	Localization	Function*
Mm STIA F1 (13)	CD45^–^PDPN^+^THY1^+^ *Sfrp2, Col11a1, Mfap4, Fap, Thy1, Col1a1, Col11a1, Col8a1, Mdk, Postn, Mmp13*	Synovial sublining	Positive regulation of osteoclast differentiation, regulation of angiogenesis, chondrocyte development, trabecule formation, Collagen metabolic process
Mm STIA F2 (13)	CD45^–^PDPN^+^THY1^+^ *Tnfaip6, Inhba, Prg4, Fap, Prg4, Hbegf, Htra1, Sema3a, Clic5, Tspan15*	Synovial sublining	Inflammatory response, cytokine production, regulation of leukocyte chemotaxis, positive regulation of defense response, fibroblast proliferation
Mm STIA F3 (13)	CD45^–^PDPN^+^THY1^+^ *Apod, C3, Cd34, Mfap5, Clip, Cxcl14*	Synovial sublining	Complement activation, vasculogenesis, glucose transport, regulation of coagulation, regulation of chemokine production
Mm STIA F4 (13)	CD45^–^PDPN^+^THY1^+^ *Top2a, Hmgb2, Cdk1*	Synovial sublining	DNA-dependent DNA replication, cell division
Mm STIA F5 (13)	CD45^–^PDPN^+^THY1^–^ *Clic5, Tspan15, Prg4, Hbegf, Htra1, Sema3a*	Synovial lining	Antigen process (MHC-I), Calcium ion transport, acid secretion, hydrogen transport, mitochondrial respiratory chain complex assembly

Proteins expressed (where available) are designated in capital letters, genes expressed in italics.

*Predicted function based on gene ontology analyses.

**TABLE 2:** Human synovial fibroblast subsets in RA.

Subset	Defining characteristics	Localization
CD34^–^THY1^+^ (44)	CD31^–^CD45^–^CD34^–^THY1^+^Cadherin-11^+/–^ IL-6, CXCL12, CCL2* *Cthrc1, Twist1, Postn, Loxl2, Pdgfr*β, *Mmp14, Tnfsf11/Tnfrsf11b*	RA synovium, unspecified
CD34^–^THY1^–^ (44)	CD31^–^CD45^–^CD34^–^THY1^–^Cadherin-11^+/–^ *Mmp1, Mmp3*	OA synovium
CD34^+^ (44)	CD31^–^CD45^–^CD34^+^THY1^+/–^Cadherin-11^+/+^ *Tnfrsf11b, Il6, Cxcl2, Ccl2*	RA and OA synovium
*PRG4*^+^ SFs (46)	*Cd55, Mmp3, Prg4, Thy1^neg^*	RA lining
*CXCL12* + SFs (46)	*Cxcl12, Ccl2, Adamts1, Thy1^low^*	RA sublining
*POSTN*^+^ SFs (46)	*Postn*, collagen genes, *Thy1*	RA sublining
*CXCL14* + SFs (46)	*Cxcl14, C3, Cd34, Aspn, Thy1*	RA sublining
CD34^+^ (SC-F1) (14)	• THY1^+^CD34^+^HLA-DR^hi^ • THY1^+^CD34^+^HLA-DR^lo^ *Cd34, C3, Fos, Pdgfr*	RA sublining OA and leukocyte-poor RA sublining
HLA-DRA^hi^(SC-F2) (14)	THY1^+^CD34^–^HLA-DR^hi^ *HLA-DRA, HLA-DRB1, HLA-DPA1, Il6, Ifi30*	RA sublining
DKK3^+^ (SC-F3) (14)	THY1^+^CD34^–^HLA-DR^lo^ *Dkk3, Cadm1, Akr1c2, Capg, Col8a2*	RA and OA sublining
CD55^+^ (SC-F4) (14)	• THY1^–^CD34^–^HLA-DR^hi^ • THY1^–^CD34^+^HLA-DR^hi^ • THY1^–^Cadherin-11^–^ • THY1^–^Cadherin-11^+^ *Itga6, Hbegf, Clic5, Htra4, Dnase1l3*	RA and OA Lining RA and OA Lining OA and leukocyte-poor RA lining OA and leukocyte-poor RA lining
Hs RA F1 (13)	FAP^+^THY1^+^HLA-DR^+^ *Dkk3, Ogn, Cd9*	Unspecified
Hs RA F2 (13)	FAP^+^THY1^+^HLA-DR^+^ *Col8a1, Aebp1, Mdk*	RA sublining**
Hs RA F3 (13)	FAP^+^THY1^+^HLA-DR^+^ *Irf1, Erg1, Junb*	Unspecified
Hs RA F4 (13)	FAP^+^THY1^–^HLA-DR^+^ *Clic5, Cd55, Hbegf*	RA lining**
Hs RA F5 (13)	FAP^+^THY1^+^HLA-DR^+^ *Cd34, C3, Apod, Clip, Cxcl14*	RA sublining**
THY1^+^HLA-DR^+^[95]	THY1^+^HLA-DR^hi^	RA sublining

Proteins expressed (where available) is designated in capital letters, genes expressed designated in italics.

*Upon stimulation with recombinant TNF.

**Predicted by cluster homology to murine subsets.

### Synovial fibroblast heterogeneity

Fibroblasts in the RA synovium exhibit vast heterogeneity and several subsets have been described that differentially contribute to RA-associated pathology in mice and humans (Tables 1, 2). Single-cell transcriptomics in the synovium of serum transfer-induced arthritis (STIA) mice revealed two anatomically distinct fibroblast activation protein-α (FAPα)^+^ subpopulations, differentiated by the expression of thymus cell antigen 1 (THY1, also known as CD90) (13). FAPα^+^THY1^+^ fibroblasts were located in the synovial sublining layer and found to mediate inflammatory responses, whereas FAPα^+^THY1^–^ fibroblasts of the lining layer exhibited a tissue destructive phenotype. Of note, inducible depletion of both subsets ameliorated experimental arthritis, whereas adoptive transfer of FAPα^+^THY1^+^ fibroblasts resulted in more severe, persistent disease. Notably, the inflammatory FAPα^+^THY1^+^ subset was enriched in RA patients compared to patients with osteoarthritis (OA). Using single-cell RNA sequencing and synovial tissue organoids, it was further suggested that the positional identity of synovial fibroblasts may be regulated by the endothelium via Notch3 signaling (42).

Three-dimensional spatial transcriptomics in synovial tissue sections from RA patients revealed substantial variation both in architectural organization as well as in cell composition (43). In synovial biopsies from seropositive RA patients, CD55^+^ lining and HLA-DR^+^ and CD34^+^ sublining fibroblasts co-localized with macrophage-rich areas, while THY1^+^ fibroblasts were in close proximity to plasma cells. Single-cell RNA sequencing in synovial biopsies from RA and OA patients identified seven fibroblast clusters characterized by distinct localization and functions (44). Among those, CD34^–^THY1^+^Cadherin-11^±^ perivascular fibroblasts were greatly expanded in RA patients, exhibiting a proliferative, invasive, proinflammatory phenotype evident by the expression of migratory response genes such as *CTHRC1, TWIST1, POSTN, LOXL2, PDGFRB* and *MMP14*, transwell matrix invasion assays, and prominent *in vitro* secretion of IL-6, CXCL12 and CCL2 upon stimulation with recombinant TNF. Additionally, paired single-cell and transposase-accessible chromatin sequencing (ATAC-seq) analysis of synovial biopsies from RA patients, combined with multiplex imaging and spatial transcriptomics, suggested that local TNF, IFN-γ or IL-1β exposure can drive FLS heterogeneity, and also indicated that functional gene expression programs in fibroblasts may be shared across tissues and diseases (45). Combining mass cytometry with transcriptomics showed that THY1(CD90)^+^HLA-DR^hi^ sublining fibroblasts were expanded in RA synovia, and attributed IL6 expression to this putative key mediator of RA pathogenesis[14]. A deconvolution analysis of published RNAseq and in-house data evaluated the contribution of synovial fibroblast subsets to the synovial pathotypes described for treatment-naïve patients with early RA (46). Interestingly, *CXCL12*^+^ and *POSTN*^+^ sublining synovial fibroblasts (SFs) constituted the most prominent fibroblast subpopulation in the fibroid, treatment-resistant RA pathotype. Comparing SFs from healthy and transgenic *hTNFtg* mice overexpressing human TNF, another study identified several homeostatic and disease-associated fibroblast subsets (47). Integrating murine data with publicly available datasets from RA patients, the study revealed conserved networks of gene regulation, while it also suggested a trajectory of transcriptional remodeling culminating in an inflammatory, destructive phenotype. Notably, a spatial transcriptomics study in RA versus spondylarthritis (SpA) patients revealed marked differences in synovial tissue composition, with SpA expressing a more fibrotic profile, enriched in mesenchymal cell signatures suggestive of higher cartilage turnover (48).

## The lung as the site of initial autoimmune responses in RA

Though synovial inflammation is a key disease characteristic, the current paradigm for RA development stands that initial autoimmunity against post-translationally modified proteins may develop in tissues outside the joints (see Figure 1). The lung has been accepted as a tissue where in genetically predisposed persons and upon environmental triggers, initial breach of immune tolerance and inflammation may take place. Smoking has – amongst the diverse environmental factors – the strongest association to RA development (25), considered to act as an epigenetic modifier inducing the citrullination of proteins in mucosal areas like the lung, and, combined with HLA-DR4 alleles contributing to ACPA formation. Several studies suggest that pulmonary involvement may be present early in the disease course of RA (49). Specifically, it has been shown that some patients with early RA have airway or parenchymal abnormalities accompanied by decreased lung function (50). Furthermore, pulmonary involvement, including ILD, abnormal high resolution computed tomography (HRCT) findings or abnormal pulmonary function tests (PFT), has been identified in early RA patients with no more than 2 years of disease duration (51). An increased frequency of interstitial abnormalities has been observed in patients with both early and longstanding RA, in contrast to bronchiolar abnormalities which have been shown to be more prominent in longstanding RA (52). Interestingly, ACPA-positive RA patients have a higher prevalence of both parenchymal and airway changes compared to ACPA-negative RA patients. HRCT revealed parenchymal lung abnormalities in ACPA-positive RA patients and further molecular studies on bronchoalveolar lavage (BAL) fluid highlighted the increased levels of ACPAs in lung compartments compared to the sera of ACPA-positive RA patients, suggesting a lung-specific production of ACPAs.

Early human studies supported the concept of the lung as the initial site of environmental-tissue interactions to induce post-translational protein modifications which may act as autoantigens in genetically susceptible individuals. In a case-control study enrolling patients with recent-onset RA, it was shown that previous smoking was dose-dependently associated with the occurrence of ACPAs, and the combination of smoking and the presence of HLA-DR shared epitope (HLA-DR SE) genes increased the risk for ACPA-positive RA (53). Notably, epidemiological studies have proposed that smoking is one of the major environmental factors associated with increased risk of developing seropositive RA even when smoking was discontinued up to 10–19 years before disease onset (54). Apart from smoking exposure (55, 56), strong gene-environment interactions were observed between HLA-SE and silica (57) and HLA-SE and textile dust (58) posing a high risk of ACPA-positive RA.

In addition to epidemiological studies, analyses of bronchoalveolar lavage (BAL) and histopathology further supported the above concept. More specifically, an enrichment of citrullinated proteins has been found in BAL cells of healthy smokers compared to healthy non-smokers. This was associated with higher expression of the peptidylarginine deiminase (PAD) 2 enzyme, which catalyzes protein citrullination. Increased expression of citrullinating enzymes was also identified in bronchial mucosal biopsies of healthy smokers (59). This study provided evidence that smoking enhances PAD2 expression with consequent generation of citrullinated proteins. Elevated lymphocyte infiltration and germinal center-like structure accumulation/formation, as well as higher immune cell activation markers have been revealed in bronchial biopsies and BAL fluids of untreated patients with early ACPA-positive RA without concomitant lung disease (27). Additionally, RF and ACPAs were found in smokers lacking evidence for RA-related joint involvement. Several studies showed the strong association between smoking, lung disease, and ACPA-positive RA, supporting that lung mucosa may be a site of ACPA generation (60). Finally, neutrophil extracellular trap formation (NETosis) has also been suggested to contribute to early immune responses and lung inflammation in RA. Specifically, ACPA presence was associated with elevated neutrophil cell counts and NET levels in the sputum of persons at risk for RA development, namely first-degree relatives (FDRs) of RA patients, supporting the hypothesis that local airway inflammation and NET formation may drive ACPA production in the lung well before any articular inflammation occurs, and may play a role in the development of RA (61).

## Pathophysiology of RA-associated ILD

Pulmonary complications are the most prevalent extra-articular manifestations in RA, clinically apparent in up-to 15% of patients, while up to 60% of patients may have subclinical disease (62), contributing significantly to increased morbidity and mortality (51). The disease is pleomorphic as it can affect distinct compartments of the lungs, including pleura, airways and their vessels, parenchyma and respiratory muscles with varying degrees of inflammation and fibrosis (63, 64). The most common form of RA-associated lung disease is ILD, a group of disorders that affect the pulmonary interstitial spaces leading to reduced lung diffusion capacity (9, 10, 65, 66). ILD develops early during RA progression and has also been associated with more severe joint disease (67), while in some cases it may even precede arthritis (68). The distribution of morphological patterns of RA-ILD as assessed by high-resolution computed tomography (HRCT) differs from the other types of collagen vascular disease (CVD)-ILD (69, 70). The most typical pattern of RA-ILD is that of usual interstitial pneumonia (UIP), which is mainly fibrotic, followed by non-specific interstitial pneumonia (NSIP) that displays both fibrotic and inflammatory manifestations or infiltrates (71). Gender differences also seem to exist among the RA-ILD patterns, with women displaying more frequently NSIP (72), while UIP has positively been associated with male gender, a previous history of smoking, as well as with older age (69, 73–76). RA- ILD patterns are correlated with diverse responses to treatments and prognosis, with retrospective studies showing that UIP is more severe and is associated with worse survival in patients with RA (77). Nevertheless, there are still a few inaccuracies in the diagnosis of RA-ILD, as the association between imaging pattern is not as clearly demonstrated in RA-ILD compared to other types of ILD, highlighting the need of establishing a standard approach to monitor lung structure and function in those patients (78).

A plethora of genetic (*MUC5*, HLA genes), environmental (smoking), demographic (older age, male sex), clinical (high titers of ACPA and RF) and drug-related risk factors were reported to contribute to the increased risk of developing ILD in the context of RA (79, 80). Nevertheless, the pathophysiological link between these risk factors and the pulmonary changes remains unclear. Recent work shows similar clinical, radiographic and genetic features (*MUC5B* rs35705950, mutations in genes involved in telomere maintenance) between certain patterns of RA-ILD, in particular UIP, and idiopathic pulmonary fibrosis (IPF), which could indicate shared pathways of pathogenesis and mechanisms of fibrosis (81). Although it is well known that the profibrotic milieu in IPF is established by complex interactions between basaloid cells, invasive fibroblasts and myofibroblasts, profibrotic monocyte-derived macrophages (82)^1^ and other immune cells (83), it is still not clear whether these findings apply to RA-ILD. There are also histopathological data suggesting that the UIP subtype in RA might be different from the UIP pattern found in IPF (69, 84, 85). It has been proposed that the driving step in RA-ILD pathogenesis is airway and/or alveolar epithelial damage in genetically susceptible individuals, in the context of exposure to environmental factors (smoking, infections and dysbiosis) (86, 87). Collagen deposition and subsequent fibrosis is favored by the production of transforming growth factor (TGF)-β and platelet-derived growth factor (PDGF) by tissue resident macrophages and dendritic cells that promote epithelial- to-mesenchymal (88), and fibroblast-to-myofibroblast transition (89). Local tissue damaged is further promoted by germinal center-like structures – called inducible bronchus-associated lymphoid tissue (iBALT) (90) – which are found in close proximity with alpha-smooth muscle actin (αSMA)^+^ cells, fibroblasts and collagen. Interestingly, the formation of these structures is not strictly induced by pulmonary inflammation, as patients with IPF do not display iBALTs, suggesting that the pulmonary pathology in RA is much more complicated. Additionally, an upregulation of IL-17RA expression in areas of fibroblastic accumulation and fibrosis was reported in biopsies from RA-ILD patients compared to either IPF or normal lung tissue, suggesting a direct role for IL-17A in lung fibrosis that may be specific to RA-ILD (91). Reduced lung diffusion capacity in a small cohort of RA patients has also been positively correlated with the levels of synovial and circulating fibrocytes (92) – a subset of bone marrow-derived cells expressing both hematopoietic and stromal cell markers which are increased in the circulation and lung biopsies of patients with IPF and ILD (93, 94). These data point out that fibrocytes could be a pathogenic link between joint and lung pathology in RA (92). Until now, studies have shown that anti-fibrotic drugs such as pirfenidone and nintedanib may slow the rate of forced vital capacity in patients with RA-ILD, in particular those with the UIP pattern (95–97). Another study supported the effectiveness of nintedanib in reducing the neutrophil-mediated fibrotic potential of human pulmonary fibroblasts (HPFs), while also reducing the levels of neutrophil extracellular traps (NETs) and soluble C5b-9 in RA-ILD patients (98). Nevertheless, the majority of the trials were underpowered (99–101) or included patients with progressive fibrosing RA-ILD (102) which does not reflect most of the patients in clinical practice. Hence, it is still not clear whether these anti-fibrotic agents could be a first-line treatment option in the management of RA-ILD (103), highlighting the need for further research on their clinical benefits and safety in RA-ILD. Collectively, a better understanding of the role of fibroblast subsets in the pathophysiology of lung involvement in RA would direct future therapeutic strategies targeting both synovial and lung inflammation.

### Fibroblasts in lung fibrosis

Within the lung, the unique localization of mesenchymal cells between the epithelium and the stroma facilitates signal transduction both in tissue development as well as in tissue injury (104) (Table 3). Following injury, the lung mesenchyme, itself comprising several different fibroblasts’ subsets, plays a significant part in maintaining a balance between epithelial repair and pathological remodeling due to aberrant activation that may occasionally lead to fibrosis. Of note, lipofibroblasts (LIFs) in murine and human lung tissue have been shown to transdifferentiate to profibrotic myofibroblasts upon nicotine (105) or hyperoxia exposure (106), as well as during bleomycin challenge in fate mapping experiments using reporter mice (107). From a molecular standpoint, lipofibroblast-to-myofibroblasts transition was dependent on TGF-β1 signaling and was attenuated upon forced activation of peroxisome proliferator-activated receptor gamma (PPARγ) in primary human lung fibroblasts. Fibroblasts isolated from IPF patients were characterized by increased invasiveness mediated by hyaluronan synthase 2 (HAS2) and the hyaluronan receptor CD44 (108). This invasiveness was reminiscent of metastatic lung adenocarcinoma fibroblasts, and pathway analysis in fibroblasts from IPF patients suggested this phenotype was mediated by ERBB2 (HER2) signaling (109). These invasive fibroblasts expressed high levels of immune checkpoint ligands *Cd274* (PDL1) and *Pdcd1lg2* (PDL2), *semaphorin 7A* (CD108), *Itga6* (CD49f) and *F3* (CD142), and functional *in vitro* experiments showed that HER2 inhibition ameliorated lung fibrosis, especially when combined with anti-PDL1 treatment. Single-cell RNA sequencing of mesenchymal cells in lung biopsies from systemic sclerosis-associated interstitial lung disease (SSc-ILD) patients and healthy controls revealed two major and one minor fibroblast subset (SPINT2^hi^, MFAP5^hi^ and WIF1^hi^, respectively) in the healthy lung (110). Those subpopulations were also identified in the SSc-ILD patients evaluated, however a distinct ACTA2 expressing and actively proliferative population of myofibroblasts was expanded in patients. The results of this study suggest that within the mesenchymal cell pool, myofibroblasts undergo the most significant phenotypic changes in systemic sclerosis, upregulating collagen-producing and profibrotic genes including but not limited to *COL10A1*, *POSTN* and *ADAM12*.

**TABLE 3:** Fibroblast subsets in the human lung in health and disease.

Subset	Defining characteristics	Condition
SPINT2^hi^ fibroblasts	*Spint2, Cd14, Lmcd1, Fgfr4, Figf*	Healthy state (110)
MFAP5^hi^ fibroblasts	*Mfap5, Cd34, Thy1, Slp1, Pla2g2a*	Healthy state (110)
WIF1^hi^ fibroblasts	*Wif1, Itga10*	Healthy state (110)
SPINT2^hi^ fibroblasts	*Spint2, Cd14, Lmcd1, Fgfr4, Figf*	SSc-ILD (110)
MFAP5^hi^ fibroblasts	*Mfap5, Cd34, Thy1, Slp1, Pla2g2a*	SSc-ILD (110)
WIF1^hi^ fibroblasts	*Wif1, Itga10*	SSc-ILD (110)
Myofibroblasts	•αSMA^+^ *Acta2* •αSMA^+^THY1*^hi^*CD34^lo^ *Acta2, Col1a1, Col1a2, Col5a2, Col3a1, Col10a1, Postn, Cthrc1, Tdo2, Adam12, Mxra5, Aldh1a3*	RA-ILD (90) SSc-ILD (110)
Invasive fibroblasts	• *Erbb2* (HER2), *Sema7a, F3, Itga6, Hmga2, Dpf3, Cd274* (PDL1), *Pdcd1lg2* (PDL2) • HAS2, CD44	IPF (109) IPF (108)
Lipofibroblasts	*Fgf10, Tcf21, Limch1, a2m, Rgcc, Apoe, Fst*	IPF (110)

Proteins expressed (where available) is designated in capital letters, genes expressed designated in italics.

This activated myofibroblast state, characterized by expression of αSMA, conferring enhanced migratory and contractile capacity, has been the focus of study in several diseases (36, 37, 110) including pancreatic cancer (111), renal fibrosis (112) and wound healing following reperfusion in myocardial infarction (113). It has been shown that fibroblasts respond to exogenous mechanical loading and cell-generated tension by increasing synthesis of extracellular matrix components, as well as by switching to an αSMA-expressing myofibroblastic phenotype (114, 115). αSMA is required for focal adhesion maturation and for the initial formation of cortical filament bundles in spreading rat lung myofibroblasts (116), thus a potential mechanotransduction circuit has been suggested. According to this, tensile forces applied to cultured RAT-2 lung cells activate MAP kinase p38 through α2β1 integrin, leading to αSMA incorporation in actin filaments which in turn enhances p38 phosphorylation in a feed-forward loop (117). As αSMA expression is regulated by TGFβ canonically via the Smad-dependent signaling pathway or non-canonically by Smad-independent signaling pathways (118, 119), it constitutes a potential target of targeted RA therapies.

Although several animal models of experimental arthritis replicate joint disease of human RA, very few of them were reported to manifest lung pathology, with a pulmonary phenotype characterized by limited fibrosis that resembles more the NSIP than the UIP pattern (120, 121). Indeed, a mixed cellular and fibrotic pulmonary phenotype characterized by infiltrating macrophages, neutrophils and lymphocyte subsets and a lower degree of fibrotic changes has been reported in the SKG mouse model (122) and also in the adjuvant-induced arthritis model in rats (123). Like the iBALT structures of RA patients with ILD, ectopic lymphoid tissue development in the lungs were identified in the K/BxN transgenic mouse model of spontaneous arthritis (124), although they were not sufficient to drive fibrosis at a steady state or upon an inflammatory challenge. So far, several reports have shown that the collagen-induced arthritis model might display a pattern that resembles that of RA-ILD which could be used to study the effect of citrullination and ACPAs on disease initiation and progression (125–127). However, pulmonary lesions spontaneously decrease after the onset of joint pathology, in contrast to humans where exacerbation of the disease occurs in most of the cases as progression of the joint disease proceeds (62, 86, 125, 126). Altogether, further effort is needed for the establishment of reliable animal models of experimental arthritis that could simulate the clinical features and the mechanisms underlying RA-ILD pathogenesis.

### Therapeutic targeting of fibroblasts

Although fibroblasts contribute substantially to inflammation and joint destruction, they are not specifically targeted by advanced treatments used in RA. This is in contrast to B- and T-cells which are specifically targeted by anti-CD20 and CTLA4-Ig, respectively. Anti-cytokine treatments like TNF inhibitors and IL-6 receptor blocker may be considered to interfere with fibroblast contribution to RA, since FLS are activated by both cytokines while they are major producers of IL-6 but not TNF. However, an elegant study in mice highlighted the importance of TNF/TNFR signaling in murine arthritis and showcased that synovial fibroblast survival and inflammatory capacity was orchestrated by the p55TNFR–IKK2–Ripk3 axis, suggesting Ikk2 targeting as a potential therapeutic target alongside TNF blockade (128). Moreover, recent elegant *in vitro* studies have shown that Jak inhibition using upadacitinib in contrast to TNF inhibition with adalimumab, reversed the induction of activated HLA-DR^+^CD90^+^ RA synovial fibroblasts by NK cell-derived IFNγ, indicating this disease-associated subpopulation as a therapeutic target of current biologics (129). As previously described, fibroblast depletion in arthritic mice ameliorated disease development, though it remains unclear how this approach could become clinically possible. Interestingly and relevant to RA-ILD pathology, in bleomycin-induced pulmonary fibrosis mouse models, the Jak inhibitor baricitinib attenuated disease severity via inhibition of the TGF-β1/non-Smad and TGF-β1/JAK/STAT signaling pathways, therefore limiting fibroblast activation and epithelial cell injury, respectively (130). Baricitinib administration in collagen-induced arthritis (CIA) on DBA/1 mice also attenuated pulmonary fibrosis by inhibiting the Jak2/Stat3 pathway, which has been found to play a key role in ILD (131).

## Conclusion

Considering the rich repository of literature published over the past few decades investigating molecular pathways and cellular networks in RA, it is becoming clear that fibroblasts constitute important players in disease pathogenesis, progression and treatment response. Both within the synovium as well as in the lung, fibroblast subsets mediate events that dramatically alter the tissue microenvironment and affect clinical outcome. Aggressive fibroblast states are associated with severe disease and their abundance has proved to hinder effective responses to treatment. At the same time, studies in experimental animals and cell cultures have offered insights into potential direct or indirect impacts of treatments on fibroblast activation, which have not yet been fully translated to inform disease management. Further, elegant studies are needed to assess the functional importance of fibroblasts implicated in RA, particularly within the lung, where our knowledge of molecular mechanisms mediating disease early stages as well as RA-ILD is very limited (see Figure 1).

Moreover, seeing as several identified fibroblast subsets may overlap to a large extent and mediate similar processes, even across organ systems and diseases, collaborative efforts need to be made in order to fully dissect complex networks of interaction, understand causal relationships and identify precise and informative biomarkers, creating knowledge that can be applied to clinical practice.

## Footnotes

1
https://www.ipfcellatlas.com/
